# Clinical Applicability of Various Treatment Approaches for Upper Gastrointestinal Submucosal Tumors

**DOI:** 10.1155/2016/9430652

**Published:** 2016-01-11

**Authors:** Jing Zhang, Kaili Huang, Shigang Ding, Ye Wang, Te Nai, Yonghui Huang, Liya Zhou

**Affiliations:** Department of Gastroenterology, Peking University Third Hospital, Beijing 100191, China

## Abstract

Submucosal tumor (SMT) is a disease that is commonly discovered during endoscopic examination. With advances in endoscopic ultrasonography (EUS) technology, this technique has become the primary screening method for the diagnosis of upper gastrointestinal SMTs. The present study summarized the clinical data of patients who were examined and diagnosed with upper gastrointestinal SMTs by EUS, underwent endoscopic therapy or surgical treatment, and received final pathological results in our hospital between January 2011 and September 2014. Our results show that endoscopic therapy has become the main approach for the treatment of upper gastrointestinal SMTs with the development and maturation of endoscopic technology in recent years. Our conclusion suggests that the selection of endoscopic methods, such as endoscopic mucosal resection (EMR), endoscopic submucosal dissection (ESD), and peroral submucosal tunneling endoscopic resection (STER), under the guidance of EUS is safe and effective for the treatment of upper gastrointestinal SMTs.

## 1. Introduction

Submucosal tumors (SMTs) are a class of protruding lesions with normal mucosa-covered surfaces. SMT is a disease that is commonly discovered during endoscopic examination. With advances in endoscopic ultrasonography (EUS) technology, this technique has become the primary screening method for the diagnosis of upper gastrointestinal SMTs. In the past, as the majority of upper gastrointestinal SMTs are benign, SMTs with diameters < 2 cm were primarily subjected to follow-up observation. If a tumor has a larger diameter or clinical symptoms have developed, the tumor is resected. With the development and maturation of endoscopic technology in recent years, endoscopic therapy has become the main approach for the treatment of upper gastrointestinal SMTs. In particular, the development and maturation of endoscopic submucosal dissection (ESD) and peroral submucosal tunneling endoscopic resection (STER) allow for the endoscopic resection of SMTs with large diameters (>2 cm) and SMTs originating from the deeper layer of the gastrointestinal tract (muscularis propria). The present study summarized the clinical data of patients who were examined and diagnosed with upper gastrointestinal SMTs by EUS, underwent endoscopic therapy or surgical treatment, and received final pathological results in our hospital between January 2011 and September 2014. In addition, the present study analyzed different treatment choices and treatment-induced complications and provides the basis for the choice of treatment of upper gastrointestinal SMTs.

## 2. Materials and Methods

### 2.1. Research Subjects

A retrospective analysis was conducted on 154 patients who (i) were examined and diagnosed with upper gastrointestinal SMTs by EUS in our hospital between January 2011 and September 2014, (ii) underwent endoscopic therapy or surgical treatment in our hospital, and (iii) received a final pathological diagnosis. The patients carried a total of 165 lesions. Among the 154 patients, 76 were male and 78 were female (male : female = 0.97 : 1). The age of the patients ranged from 17 to 78 years with a mean age of 52.82 years. All of the patients who underwent endoscopic examination and treatment signed informed consent document. The present study was approved by the Ethics Committee of Peking University Third Hospital.

### 2.2. Research Methods

#### 2.2.1. Data Collection

All of the patients were subjected to EUS examination. All of the EUS procedures were performed by experienced physician endoscopists in the Department of Gastroenterology. The following data were collected: basic information of the patients (i.e., name, gender, age, hospital admission number, pathology number, and examination number), the diagnostic results of the EUS examination, the utilized treatment methods, any postoperative complications, and the results of postoperative pathologic diagnosis.

#### 2.2.2. Instruments

The EUS system used in the present study consisted of a FUJINON EG530UR scope, a Fujinon SU-8000 ultrasound processor, and a SP-702 sonoprobe system (miniprobe EUS system). The utilized gastroscopes included the FUJINON EG590, FUJINON EG410, FUJINON EG450, FUJINON EC450WM5, and the FUJINON EC410. Other equipment included FD-410LR hemostatic forceps (Olympus, Japan), a KD-611L IT knife (Olympus, Japan), a KD-620L hook knife (Olympus, Japan), a ERBE200 high-frequency electrosurgical generator (ERBE Elektromedizin GmbH, Germany), and an APC300 argon plasma coagulation (APC) unit (ERBE Elektromedizin GmbH, Germany).

#### 2.2.3. Treatment Methods

*(1) Endoscopic Mucosal Resection (EMR)*. The target lesion was lifted by injection of appropriate amounts of saline-epinephrine solution (0.005% epinephrine) into the surrounding area using a submucosal injection needle. The entire lesion was then trapped with a snare. Subsequently, the snare was tightened at the base of the lesion, and the lesion was resected using a high-frequency electric current. Immediately after removal, the specimens were fixed with 4% formaldehyde and sent for histopathological examination. 

*(2) ESD*. After marking the resection border using argon cautery, a proper volume of a saline solution containing 0.005% epinephrine and methylene blue was injected into the base of the lesion in order to raise it. The mucosa surrounding the lesion was precut using a needle knife with a bent tip. The submucosal connective tissue beneath the lesion was peeled off, and the lesion was completely resected. Subsequently, electrocoagulation and APC were performed to stop bleeding, and titanium clips were applied to close the wound. The specimens were fixed with 4% formaldehyde immediately after removal and sent for histopathological examination.

*(3) Peroral STER*. Under an endoscope, a solution of indigo carmine, epinephrine, and normal saline was injected into an area approximately 5 cm from the upper margin of the tumor. A longitudinal incision 1.5–2.0 cm in length was created in esophageal or gastric mucosa, and the submucosal layer was separated out. A transparent cap was attached to distal end of the endoscope, which allowed the entry of the endoscope into the submucosal layer. A submucosal tunnel was artificially established along the submucosa until the tunnel extended 1-2 cm beyond the tumor, leaving the tumor completely exposed. The tumor was separated from the surrounding muscular layers with a hook knife and an IT knife and then excised from the muscularis propria layer. The integrity of the tumor and tumor capsule were well maintained during the process. After removal of the specimen, the wound was washed with a copious volume of sterile saline. In the case of bleeding, electrocoagulation or APC cauterization was employed to stop the bleeding. Lastly, metal clips were applied to suture the mucosal incision (entrance of the tunnel).

*(4) Endoscopic Submucosal Excavation (ESE)*. The margin of the protruded lesion was accurately marked by electrocoagulation using a needle knife or APC. A solution of indigo carmine, epinephrine, and normal saline was injected submucosally into multiple sites at the margin of the elevated lesion. The mucosa was then incised along the marked points using a hook knife or an IT knife, and the lesion was resected along its capsule using a hook knife and an IT knife. After removal of the lesion, electrocoagulation or APC was performed to cauterize the visible small blood vessels in the wound. The wound was closed with metal hemostatic clips when necessary.

*(5) Surgical Procedures*. This included endoscopic (Laparoscopic and Thoracoscopic) surgery and laparotomy.

### 2.3. Statistical Analysis

The data were subjected to statistical analysis using SPSS 19.0 software. Comparisons between two groups of samples were performed using Pearson's chi-square test. *P* values < 0.05 were considered statistically significant.

## 3. Results

### 3.1. General Information

The 154 patients harbored a total of 165 lesions. Among the 154 patients, 2 patients harbored 1 lesion in the esophagus and 1 lesion in the stomach, 4 patients carried 2 lesions in the esophagus, 1 patient developed 3 lesions in the stomach, and 1 patient had 4 lesions in the stomach. The age of the patients ranged from 17 to 78 years, with a mean age of 52.82 years. The incidence rate was highest among patients aged 50–59 years (35.1%), followed by patients aged 60–69 years (20.8%). The long diameter and location of 164 lesions (the measured value for the long diameter of one gastric lesion was not available) were analyzed, and the results are shown in Table 1. The minimum value of the long diameter was 0.3 cm, and the corresponding lesion was located in the esophagus. The maximum value of the long diameter was 6.2 cm, and the corresponding lesion was located in the stomach. The average long diameter was 1.8 cm.

Among the 165 lesions, 81 were esophageal, 79 were gastric, and 5 were duodenal, accounting for 49.1%, 47.9%, and 3.0% of the total lesions, respectively. Among the 81 esophageal lesions, the highest incidence rate (23.6%) was observed for lesions in the middle part of the esophagus, accounting for 48.1% (39/81) of all esophageal lesions. Among the 79 gastric lesions, the highest incidence rate (16.4%) was observed for lesions in the gastric body, followed by the lesions in the gastric antrum (13.9%). Lesions in the gastric body and gastric antrum accounted for 34.1% (27/79) and 29.1% (23/79) of all of the gastric lesions, respectively. In terms of the 5 duodenal lesions, 2 were located in the duodenal bulb, 1 was located at the junction between the duodenal bulb and the descending duodenum, and 2 were located in the descending part of the duodenum.

The originating layers of the lesions were analyzed. It was found that, among the 165 lesions, the largest percentage (76 lesions, 46.1%) originated from the 2nd layer of the gastrointestinal tract (muscularis mucosa), followed by the 4th layer (muscularis propria) (60 lesions, 36.4%). One lesion originated from the 5th layer (serosa). Postoperative pathological analysis indicated that the lesion was a gastrointestinal stromal tumor.

### 3.2. Analysis of the Treatments

**Figure 1:**
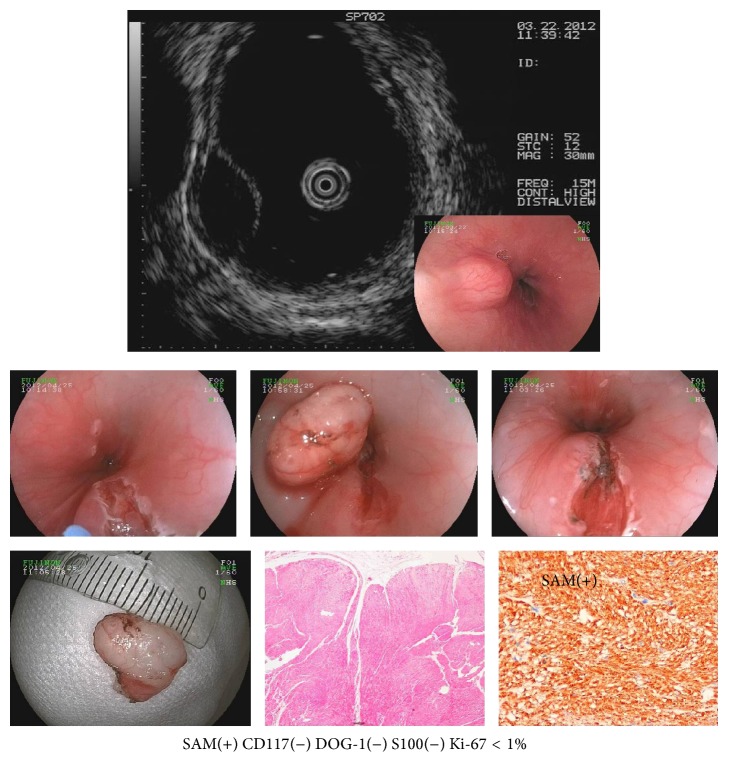
Esophageal leiomyoma.

**Figure 2 fig2:**
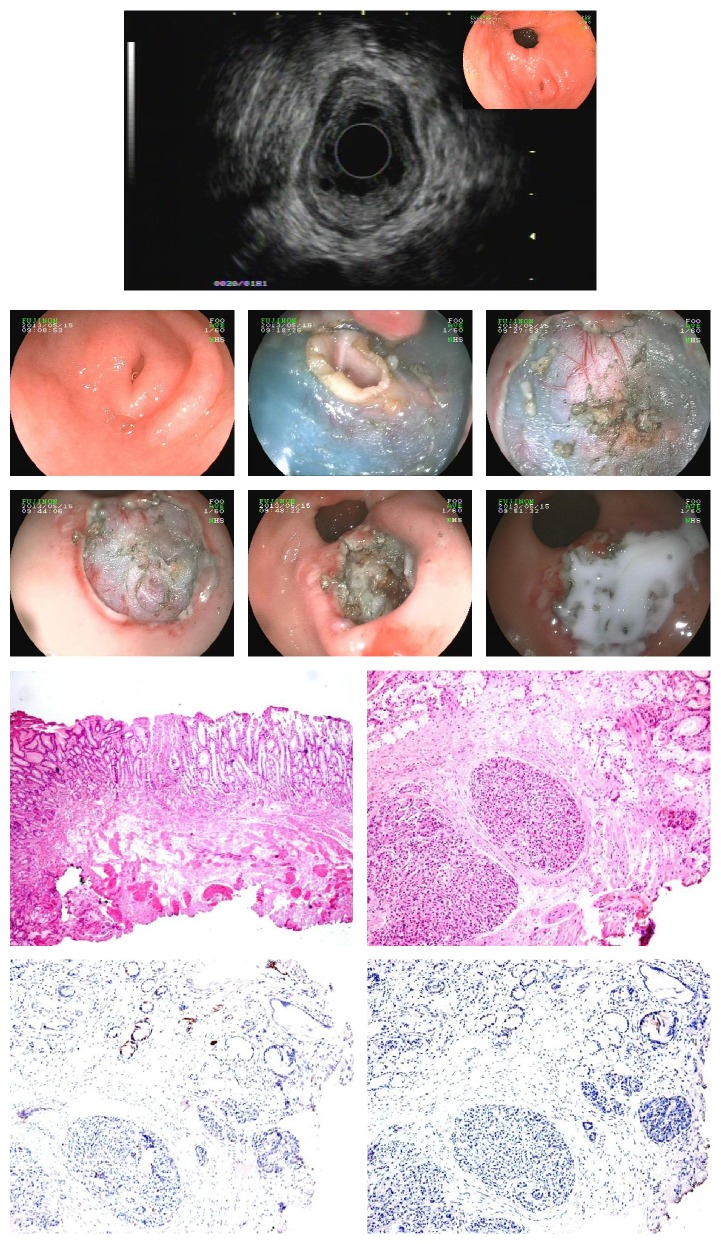
Heterotopic pancreas in the gastric antrum.

**Figure 3:**
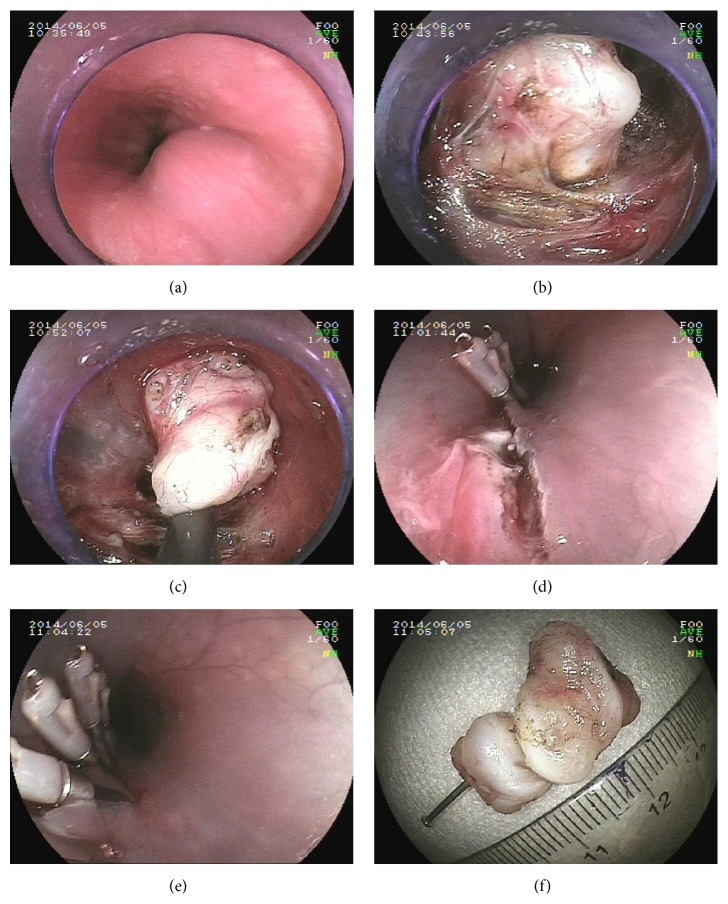
STER procedure for the resection of esophageal leiomyoma. (a) Endoscopic examination of esophageal leiomyoma. Diameter of the tumor: 2.5 cm. (b) Establishment of the submucosal tunnel. (c) Resection of the tumor. (d) and (e) Closure of the entrance of the submucosal tunnel. (f) Fixation of the specimens.

The methods employed to treat SMTs can be divided into endoscopic therapy (used to treat 102 lesions) and surgical intervention (used to treat 63 lesions). Endoscopic therapy included EMR (41 lesions), ESD (44 lesions), STER (14 lesions), and ESE (3 lesions) (Figures 1 [Fig fig2]–3). Surgical treatments included endoscopic surgery (laparoscopic surgery was employed to treat gastric and duodenal lesions, while thoracoscopic surgery was employed to treat esophageal lesions) and laparotomy. Among the 63 patients who received surgical treatment, 55 received endoscopic surgery, while 8 received laparotomy (all 8 patients were originally candidates for endoscopic surgery but required conversion to laparotomy due to intraoperative findings of severe abdominal adhesions during the laparoscopic procedure). More specifically, 59 of the 63 patients underwent local tumor excision (local excision of esophageal and duodenal SMTs and wedge resection of gastric SMTs), 2 underwent open subtotal gastrectomy, 1 received open radical total gastrectomy, and 1 received laparoscopic proximal gastrectomy.

The medical information of the patients who received endoscopic therapy or surgical treatment was analyzed. No significant difference existed with respect to the age range of the patients. The mean ages of the endoscopically treated patients and the surgically treated patients were 51 years and 53 years, respectively.

#### 3.2.1. Pathological Diagnosis after Resection of Lesions and Treatment Methods

See Table 2.

#### 3.2.2. The Originating Layers of the Tumors Were Analyzed in Patients with Endoscopic Treatment and the Surgically Treated Patients

The resulting two sets of data were subjected to the *χ* ^2^ test. The *χ* ^2^ value was 86.308, and the *P* value was <0.05, indicating that the difference was statistically significant. The data for the cases were compared in detail. It was found that endoscopic therapy was the most common treatment selected to treat lesions originating from the 2nd and 3rd EUS layers (70/76 and 22/28, 92.1% and 78.6%, resp.), whereas endoscopic surgery was the most frequent treatment used to treat lesions derived from the 4th EUS layer (43/60, 71.7%) (Table 3).

#### 3.2.3. Tumor Size Analysis

As shown in Table 3, the diameters of the tumors in the endoscopically treated group ranged from 0.31 cm to 4 cm (mean diameter: 1.12 cm), whereas the diameters of the tumors in the surgically treated group ranged from 0.67 cm to 6.2 cm (mean diameter: 2.88 cm). Endoscopic therapy was the most frequent treatment selected to treat lesions < 2 cm in diameter (92/104, 85.5%), while surgery was the most frequent treatment selected to treat lesions with diameters ≥ 2 cm (50/60, 83.3%). The group of tumors with long diameter < 2 cm was compared to the group of tumors with long diameters ≥ 2 cm, and the number of endoscopically treated lesions (102 lesions) was compared to the number of surgically treated lesions (62 lesions). The chi-square test was performed, and the *P* value was <0.05, indicating that the difference was statistically significant. The results showed that the diameter of the lesions (specifically whether the diameter was >2 cm) might serve as a reference for selection of treatment methods (surgery or endoscopic therapy) (Table 4).

#### 3.2.4. The Location of Tumors Was Analyzed in the Endoscopically Treated Patients and the Surgically Treated Patients

The two sets of data were subjected to the *χ* ^2^ test based on tumor locations. The *χ* ^2^ value was 46.2, and the *P* value was <0.05, indicating that the difference was statistically significant. The data of the cases were further compared in detail. It was found that endoscopic therapy was the most frequently selected to treat esophageal SMTs (71 tumors), whereas surgery was the most frequently selected treatment for gastric SMTs (51 tumors) (Table 4).

#### 3.2.5. Lesion Locations and Endoscopic Approaches Were Subjected to Further Detailed Analysis, and the Results Are Shown in Table 5

[Table tab5]

With respect to tumor locations, EMR was the most commonly used treatment for esophageal lesions (39/41), ESD was the most common treatment for lesions in the gastric antrum (15/44), STER was the most common treatment for lesions in the middle part of the esophagus (6/14) and the gastric cardia (4/14), and surgery was the most common treatment for lesions in the gastric body (24/63). The endoscopic treatment techniques employed in the present study were compared, and the detailed data shown in Table 5 were comprehensively analyzed. From these results, it can be concluded that as the size of an SMT increases and the layer from which the tumor originates becomes deeper, the chosen treatment regimen gradually changes from EMR to ESD and then to STER.

### 3.3. Treatment-Induced Complications

Among the 102 patients who received endoscopic therapy, 3 patients (2.9%) developed complications. One patient suffered post-ESD bleeding, one patient suffered post-ESD perforation, and one patient developed mediastinal emphysema after STER. The complications resolved after conservative treatment.

Among the 63 patients who underwent surgical intervention, 2 patients (3.2%) developed complications. Vomiting occurred in 1 patient at 5 d after open subtotal gastrectomy. Considering that the patient developed gastrojejunal anastomotic edema and stenosis, jejunal feeding tube placement and conservative treatment were administered. The other patient suffered upper respiratory tract infection and was given conservative treatment. The conditions of both patients improved after the treatments.

## 4. Discussion

SMTs refer to a class of gastrointestinal lesions that originate below the mucosal layer (primarily from the muscularis mucosa, submucosa, and muscularis propria). SMTs mainly include leiomyoma, stromal tumors, lipomas, and neurogenic tumors. Patients with SMT usually display no specific symptoms. In most cases, SMTs are discovered incidentally during endoscopic examination. Gastrointestinal SMTs are usually benign, and only a small portion are malignant, primarily including leiomyosarcomas, liposarcomas, and malignant stromal tumors. The majority of gastrointestinal SMTs have no specific endoscopic appearance. Therefore, it is very difficult to determinate the nature of SMTs using an ordinary endoscopic examination. EUS is capable of determining, in general, the nature of a lesion based on the originating layer, size, and internal echoes of the lesion. Therefore, EUS may assist in both diagnosis and guiding treatment [1, 2]. At present, international guidelines recommend close clinical follow-up for SMTs < 2 cm in diameter. Surgical resection is commonly conducted on SMTs > 2 cm in diameter. However, long-term follow-up not only places a very large financial burden on patients but also causes severe psychological stress. Traditional surgical excision results in large surgical wounds and slow recovery, which have a negative impact on the quality of life of the patients.

EMR is the first technology for the treatment of gastrointestinal SMTs. Previously, EMR was only employed to treat lesions confined to the mucosa, such as polyps. With advances in technology and improvements in instruments, the indications for EMR have expanded to submucosal lesions. EMR is suitable for treating SMTs that originate from the superficial layers and which have a diameter between 1 and 2 cm. In terms of the originating layer of the lesions, the absolute indications for EMR are lesions that originate from the m1 and m2 layers, while the relative indications include lesions that originate from the m3 and sm1 layers. If the mucosal layer becomes separated from the muscularis propria and a local uplift of the mucosa occurs after local injection, EMR may be chosen to treat the lesions. With the invention of the IT knife, ESD began to be used to excise early gastrointestinal cancer and precancerous lesions larger than 2 cm in diameter. ESD allows a one-time, complete en bloc resection of the lesions and provides materials for accurate final pathological diagnosis, fully reflecting the superiority of endoscopic minimally invasive resection. After ESD, there are no marginal tissue residues, and the recurrence rate is rather low. ESD is suitable for treating superficially located SMTs (in the muscularis mucosa or submucosa) larger than 2 cm in diameter and SMTs with wide bases [3, 4]. Under rare circumstances, ESD is employed to treat lesions in the muscularis propria. However, the indications for ESD do not include such lesions. Treatment of lesions involving the muscularis propria with ESD increases the risk of perforation and bleeding. A technique named ESE was later developed to allow resection of lesions that originate from the muscularis propria (a more complete discussion of the ESE technique is included in later sections of the present paper). However, cautious operation is necessary. According to previous reports, the rate of ESD-induced perforation is approximately 4% and the bleeding rate is <5%. In case of bleeding, electric coagulation is performed and hemostatic agents are sprayed onto the wound. If necessary, titanium clips are used to clamp the bleeding vessels. In addition, the patients are prescribed certain treatments, such as fasting, water deprivation, fluid infusion, and acid suppression. Generally, the bleeding can be successfully stopped after the above treatments [3, 4]. Chinese scholars have creatively developed several new endoscopic resection techniques on the basis of ESD therapy and applied these new techniques to treat gastrointestinal SMTs. Such novel techniques allow for accurate diagnosis of the lesions and are able to achieve therapeutic goals. The new techniques include the following. (1) ESE: with the wide application of ESD in clinical practice and the continuous maturation of ESD technique, Zhou et al. [5] gradually extended the technique and was the first in the world to use it for gastrointestinal SMTs resection. To distinguish ESD as applied to treat gastrointestinal muscularis mucosa-derived tumors from its use to treat gastrointestinal SMTs, the terminology “ESE” was coined to describe the technique in which various knives are used to excise SMTs originating from the muscularis propria. As an extension of ESD, ESE provides a new approach for the treatment of gastrointestinal SMTs that originate from the muscularis propria. (2) STER: the removal of SMTs that originate from the muscularis propria may require a full-thickness excision, resulting in perforation. Most perforations can be successfully repaired using endoscopic methods. However, in the case of significant gastrointestinal wall loss, it is rather different to achieve tight wound sealing during surgery. Therefore, some patients may suffer postoperative gastrointestinal leakage and secondary infection of the thoracic or peritoneal cavity. The endoscopic tunnel technology peroral endoscopic myotomy (POEM) was initially used to treat cardiac achalasia [6]. Xu et al. first proposed the application of this technology in the resection of SMTs, and this technique was termed STER [7]. Initially, STER was primarily used to treat SMTs originating from the muscularis propria, with a diameter < 3.5 cm, and located in the middle or lower portion of the esophagus. However, the technique has matured, and there are no longer “forbidden zones” with respect to tumor location and size. STER has been used to treat tumors located in the upper esophagus and stomach as well as tumors > 3.5 cm in diameter. STER differs not only from traditional endoscopic therapy, which is limited to the gastrointestinal lumen, but also from endoscopic therapy performed outside of the gastrointestinal tract lumen through a natural orifice. STER cleverly takes advantage of the space between the gastrointestinal mucosal and muscular layers. In STER, tumors are completely resected while the integrity of the gastrointestinal tract is maintained, thereby avoiding or greatly reducing the incidence of gastrointestinal leakage and secondary infection in the thoracic and peritoneal cavities.

The present study summarized the criteria for selecting different resection methods. The originating layer of the tumor serves as one of the criteria. In general, endoscopic therapy was the most frequently selected treatment for superficially located tumors (the 2nd and 3rd layer), while surgical intervention was often chosen for tumors originating from the deeper layers (the 4th layer). The diameters of the lesions also affect method selection. The present study divided the lesions into different groups based on diameter (>2 cm or <2 cm). The results indicated that surgical resection were often selected for tumors > 2 cm in diameter, while endoscopic procedures were the most frequently selected treatment for tumors < 2 cm in diameter. We have reviewed the literature, and Japanese and European guidelines currently recommend surgery for tumors > 5 cm in diameter and EUS-guided fine needle aspiration (EUS-FNA) for tumors with diameters between 3 and 5 cm. However, if the tumor is a gastrointestinal stromal tumor (GIST), surgery is recommended. If the tumor is leiomyoma or Schwannoma, intensive follow-up is recommended. The Japanese and European guidelines provide the following recommendations in regard to tumors < 3 cm in diameter: conducting endoscopic therapy such as EMR, performing EUS-FNA to obtain pathological data, or conducting follow-up observation (follow-up is recommended for GISTs only if the diameter is <2 cm) [1, 2]. The results of the present study are similar to those reported in the Chinese literature. Surgical procedures are often selected to treat SMTs that are >2 cm in diameter and originate from deeper layers. However, with the rapid development and continuous maturation of endoscopic techniques, the size, location, and nature of the lesion are all factors to be considered in the determination of the indications for endoscopic therapy. However, the originating EUS layer of the lesion is perceived as a more important criterion for selection of the treatment methods. Particularly since the development of techniques such as ESD and STER, the endoscopic resection of tumors >2 cm in diameter has become realizable.

In the present study, complications only occurred after endoscopic treatment of 3 lesions (2.9%), and these complications were resolved by conservative treatment. The findings therefore indicate that endoscopic therapy is safe.

The present study retrospectively summarized SMT cases in China that were diagnosed using EUS and treated by various methods, including a variety of endoscopic procedures. However, the present study has its own limitations. First, all cases that were retrospectively analyzed in the present study were confined to 1 hospital. In addition, there were no clearly defined criteria for the selection of treatment methods. Physician endoscopists chose various treatment approaches based on their own experience. A prospective study needs to be performed to further define the criteria for the selection of various treatments for different lesions. The present study included a rather large number of SMT cases and provides guidance with respect to the selection of SMT treatments. In summary, the selection of endoscopic therapies, such as EMR, ESD, and STER, under the guidance of EUS is safe and effective for the treatment of SMTs.

## Figures and Tables

**Table 1:** The long diameter and location of the lesions.

	Esophagus	Stomach	Duodenum
Long diameter (cm)			
Minimum value	0.3	0.6	1
Maximum value	6	6.2	2.8
Mean value	1.28	2.38	1.64
Number of lesions < 2 cm	69	32	3
Number of lesions ≥ 2 cm	12	46	2

**Table 2:** Pathological diagnosis after resection of lesions and treatment methods.

Pathological diagnosis	EMR	ESD	STER	ESE	Endoscopic surgery	Laparotomy	Total
Stromal tumor		2	4	2	36	5	49
Leiomyoma	34	22	10	1	7		74
Heterotopic pancreas		9			4	1	14
Lipoma	1	5			1		7
Carcinoid		1				2	3
Brunner adenoma	1	1					2
Schwannoma	1				2		3
Cyst		1			1		2
Granulosa cell tumor	2						2
Bronchogenic cyst					1		1
Fibrous polyp		1					1
Spindle cell tumor					1		1
Hemangioma					1		1
Hyperplastic polyps		1					1
Amyloidosis	1						1
High density-induced compressional deformation of spindle cells	1						1
Benign gastric lesions that display significant hyperplasia of vascular fibrous tissue and hyaline degeneration					1		1
Chronic inflammation with intestinal metaplasia; irregularly arranged smooth muscle tissue and hyperplastic fibrous tissue lying beneath the mucosa		1					1
Total	41	44	14	3	55	8	165

**Table 3:** Comparison of the originating layers of SMTs and treatment choices.

	2nd layer	3rd layer	4th layer	5th layer	Total
Endoscopic treatment	70	22	10	0	102
Surgical treatment	6	6	50	1	63
Total	76	28	60	1	165

*χ* ^2^	86.308				
*P* value	<0.05				

**Table 4:** Comparison of the locations and sizes of SMTs and the treatment choices.

	Age (years)			Location (number of SMTs)			Diameter (cm)			<2 cm (number of SMTs)	≥2 cm (number of SMTs)
	Min.	Max.	Mean	Esophagus	Stomach	Duodenum	Min.	Max.	Mean		
Endoscopic treatment	17	78	51	71	28	3	0.31	4	1.12	92	10

Surgical treatment	18	78	53	10	51	2	0.67	6.2	2.88	12	50

*χ* ^2^ test				46.20						83.41	
*P* value				<0.05						<0.05	

**Table 5 tab5:** Detailed analysis of the locations, originating layers, and long diameters of the tumors and the corresponding treatment choices.

		EMR	ESD	STER	ESE	Endoscopic surgery	Laparotomy	Total
Esophagus	Upper part	10	7	0	0	0	0	17
	Middle part	19	9	6	0	5	0	39
	Lower part	10	8	2	0	5	0	25
	Total	39	24	8	0	10	0	81

Stomach	Gastric cardia	0	1	4	0	5	0	10
	Gastric fundus	0	1	0	2	6	2	11
	Junction between gastric body and fundus	0	0	1	0	3	0	4
	Gastric body	0	1	1	1	21	3	27
	Gastric transitional zone	0	1	0	0	3	0	4
	Gastric antrum	0	15	0	0	6	2	23
	Total	0	19	6	3	44	7	79

Duodenum	Duodenal bulb	1	0	0	0	0	1	2
	Junction between duodenal bulb and descending duodenum	0	0	0	0	1	0	1
	Descending duodenum	1	1	0	0	0	0	2
	Total	2	1	0	0	1	1	5

Originating layer	2nd layer	41	23	6	0	6	0	76
	3rd layer	0	20	1	1	5	1	28
	4th layer	0	1	7	2	43	7	60
	5th layer	0	0	0	0	1	0	1

Long diameter (cm)	Minimum value	0.31	0.4	0.78	1.1	0.67	1.2	
	Maximum value	3.5	4	2.47	1.8	6	6.2	
	Mean value	0.69	1.43	1.34	1.42	2.89	2.79	

Number of lesions < 2 cm		40	36	13	3	10	2	104
Number of lesions ≥ 2 cm		1	8	1	0	44	6	60

Total		41	44	14	3	54 (+1)	8	165
